# How gastrin-releasing peptide receptor (GRPR) and α_v_β_3_ integrin expression reflect reorganization features of tumors after hyperthermia treatments

**DOI:** 10.1038/s41598-017-06100-7

**Published:** 2017-07-31

**Authors:** Sandra Hallasch, Sindy Frick, Maximilian Jung, Ingrid Hilger

**Affiliations:** 1 Department of Experimental Radiology, Institute for Diagnostic and Interventional Radiology, Jena University Hospital – Friedrich Schiller University Jena, Am Klinikum 1, D-07747 Jena, Germany; 2 Department of Medical Engineering and Biotechnology, University of Applied Science Jena, Carl-Zeiss Promenade 2, 07745 Jena, Germany

## Abstract

The outcome of tumor treatment via hyperthermia in the clinic has been reported to be heterogeneous. Here, we assessed how the presence of gastrin-releasing peptide receptor (GRPR) and α_v_β_3_ integrin together with the morphology of the vascularization reflects the growth behavior of tumors after hyperthermia treatment. MDA-MB-231 tumor bearing mice were treated either with high (46 °C) or low dose (42 °C) water hyperthermia for 60 min. Changes of GRPR and α_v_β_3_ integrin expression were assessed via multiplexed optical imaging. Vascularization was reconstructed and quantified by µCT imaging after contrast agent injection. We found that high dose hyperthermia is capable of increasing the expression of GRPR, α_v_β_3_ integrin, CD31, and Ki67 in tumors. Also the morphology of tumor vasculature changed (increased relative blood volume and small-diameter vessel density, decreased expression of α-SMA). Low dose hyperthermia induced comparatively moderate effects on the investigated protein expression pattern and vascular remodeling. We conclude that under defined circumstances, specific temperature doses affect the reorganization of tumor regrowth, which is triggered by residual “dormant” cells even though tumor volumes are transiently decreasing. Further on, GRPR, α_v_β_3_ integrin expression are versatile tools to surveil potential tumor regrow during therapy, beyond the conventional determination of tumor volumes.

## Introduction

Hyperthermia has been recognized as a potent therapeutic strategy against cancer. Despite of it, its transfer and implementation into the clinical situation progresses slowly. Namely, the current clinical practice confines hyperthermia as a supportive modality, which is used in combination with other therapy regimes^1, 2^. Examples are the sensitization of cells to ionizing radiation or chemotherapeutic agents via hyperthermia^3, 4^, the hyperthermic perfusion combined with surgery or chemotherapy in case of peritoneal metastases^5^, and a general sensitization of tumor cells for subsequent chemotherapy sessions. As a single therapeutic modality the therapeutic outcome of hyperthermia was reported to be quite heterogeneous^1, 6^, particularly when external heating sources (hot air, water, infrared radiation) are used.

To the best of our knowledge, there is no consistent understanding of the biomedical reasons which are responsible for the heterogeneity of the therapy success of hyperthermia. Such investigations will require the utilization of specific protein structures, which allow the monitoring of the impact of hyperthermia on the target tumor tissue.

Importantly, the gastrin-releasing peptide receptor (GRPR) is expressed in multiple cancers such as colon^7, 8^, prostate^9^, lung carcinoma^10, 11^ breast cancer^12^, etc. GRPR has been extensively targeted with radiotracers in the preclinical and the preclinical level using bombesin (BBN, the ligand BBN is a 14-amino-acid neuropeptide)^13^ analogues in radioactive or optical probes^14, 15^. Its widespread presence in many tumors and its intensive use in imaging approaches on the molecular level make the GRPR a very attractive marker for monitoring of the tumor therapeutic efficiency of hyperthermia.

A further molecular marker which has also been widely recognized to be expressed in tumors is α_v_β_3_ integrin. In particular, in whole body imaging applications α_v_β_3_ integrin is capable of assessing the presence of tumor vascularity^16–18^, particularly in post-therapy monitoring applications^19^. Namely, α_v_β_3_ integrin is present in activated endothelial cells of newly formed vasculature in tumors. The presence of vascularity is essential for tumor growth and metastasis. For this reason, α_v_β_3_ integrin is an appealing marker for monitoring the effects of hyperthermia on vascularity.

In general, the particular behavior of GRPR and α_v_β_3_ integrin after hyperthermia treatment *in vivo* is not known so far. Therefore, we sought to address GRPR and α_v_β_3_ integrin in tumors in a multiplexed whole body-imaging approach in laboratory animals in order to evaluate the effect of hyperthermia at different temperatures and asked the following questions: First, which effects do different temperatures exert on GRPR expression and cell viability? Second, how does high or low dose hyperthermia influence the expression of α_v_β_3_ integrin and GRPR in a breast cancer xenograft? Third, to what extent does hyperthermia alter the intratumoral vessel density and relative blood volume? The knowledge of these relationships would allow a better understanding of the underlying biomedical processes and consequently a refinement of the tumor therapeutic strategies.

## Results

### Temperatures influence gastrin-releasing peptide receptor (GRPR) expression in MDA-MB-231 cells

**Figure 1:**
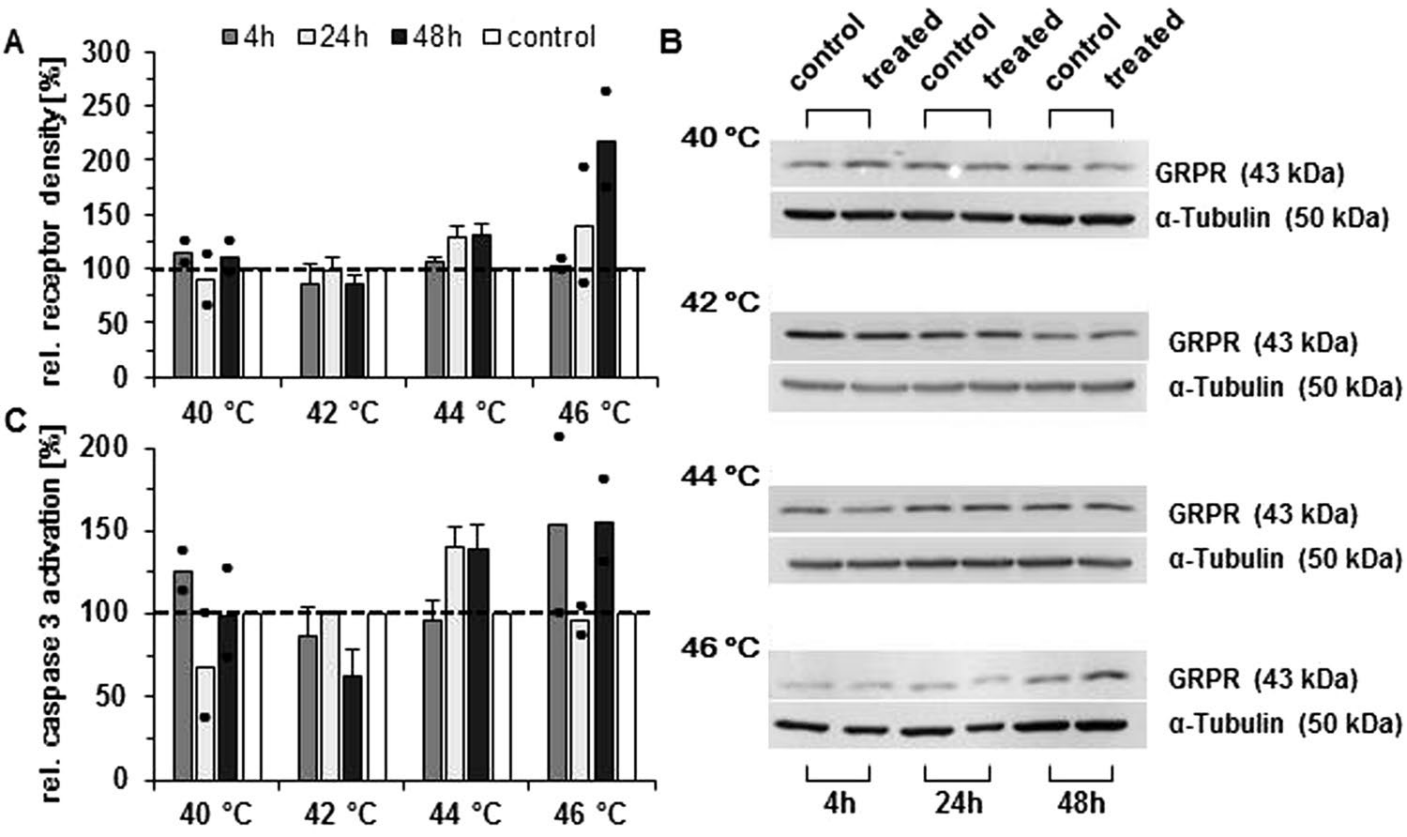
Extrinsic (hot air) hyperthermia enhances GRPR expression in MDA-MB-231 cells and this expression is associated with activation of apoptosis effector protein caspase-3. (**A**) Alteration of GRPR expression at 1 h post hyperthermia as detected by flow cytometry. (**B**) Representative Western blots for GRPR expression (1 h post hyperthermia). Full-length blots are presented in Supplementary Figure 3. (**C**) Relative proportion of cleaved and thereby active caspase-3 measured by flow cytometry at 48 h post hyperthermia. The measured values were normalized to untreated control cells. Data represent the mean ± SD with n = 3. Dots indicate the measured values if the experiments were carried out twice. For controls see Supplementary Figures 2 to 4.

Flow cytometry analysis showed that GRPR expression was enhanced in cells treated with hyperthermia at 44 °C (24 h post therapy) and increased even more when temperatures increased to 46 °C (high dose hyperthermia) (flow cytometry, Fig. 1A). At temperatures between 40 and 42 °C, no obvious changes in GRPR expression occurred. A similar situation was encountered via protein analysis (immunoblotting Fig. 1B): After hyperthermia at temperatures ranging between 40 and 44 °C, no distinct changes were found in GRPR expression between treated and untreated cells. After highly dosed hyperthermia (46 °C), the GRPR expression distinctly increased with increasing post treatment times (48 h) compared to non-treated control cells. Particularly at higher temperatures (44 and 46 °C), a tendency for increased presence of cleaved caspase-3 was observed (48 h post hyperthermia, Fig. 1C).

### Hyperthermia influences α_v_β_3_ integrin and GRPR expression in MDA-MB-231 tumors *in vivo*

**Figure 2:**
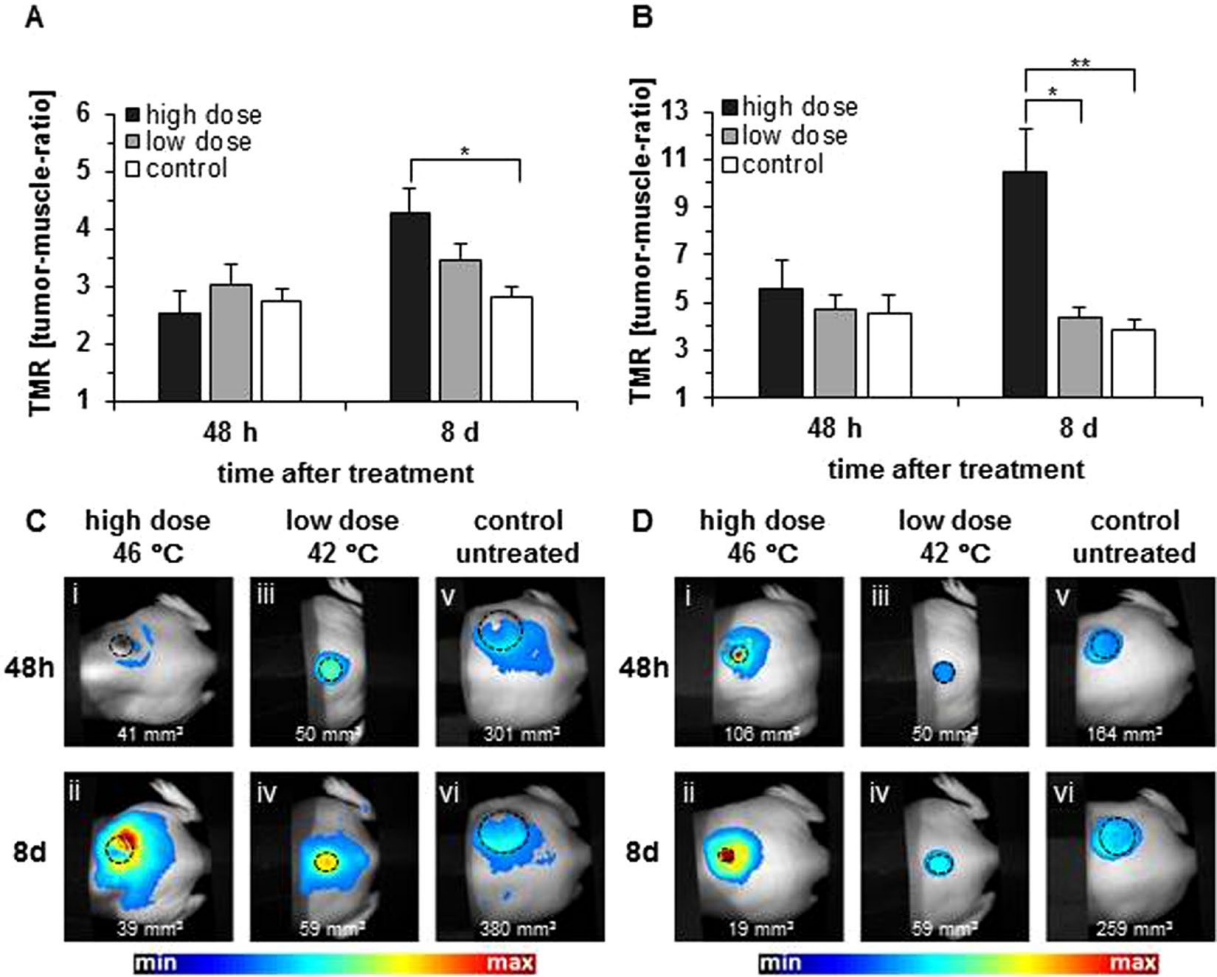
Signal intensities for GRPR and α_v_β_3_ integrin increase after hyperthermia treatment in a period of 8 days. (**A**) Tumor-to-muscle ratios (TMR) of the temperature-dependent fluorescence intensities induced by the optical probe BombesinRSense™ 680 (2 nmol). (**B**) Semi-quantitative analysis of fluorescence intensities induced by the optical probe IRDye^®^ 800CW RGD (1 nmol) at tumor areas (see methods). (**C**) Representative macroscopic images with color-coded fluorescence intensities of GRPR expression. (**D**) Representative macroscopic images with color-coded fluorescence intensities of α_v_β_3_ integrin expression. Tumor volumes at the bottom of each picture; marked tumors in black. Controls were not subjected to any hyperthermia treatment. All data represent the mean ± SEM (n = 10 in control group, n = 9 in low dose, n = 10 in high dose hyperthermia; *p ≤ 0.05 **p ≤ 0.01).

**Figure 3:**
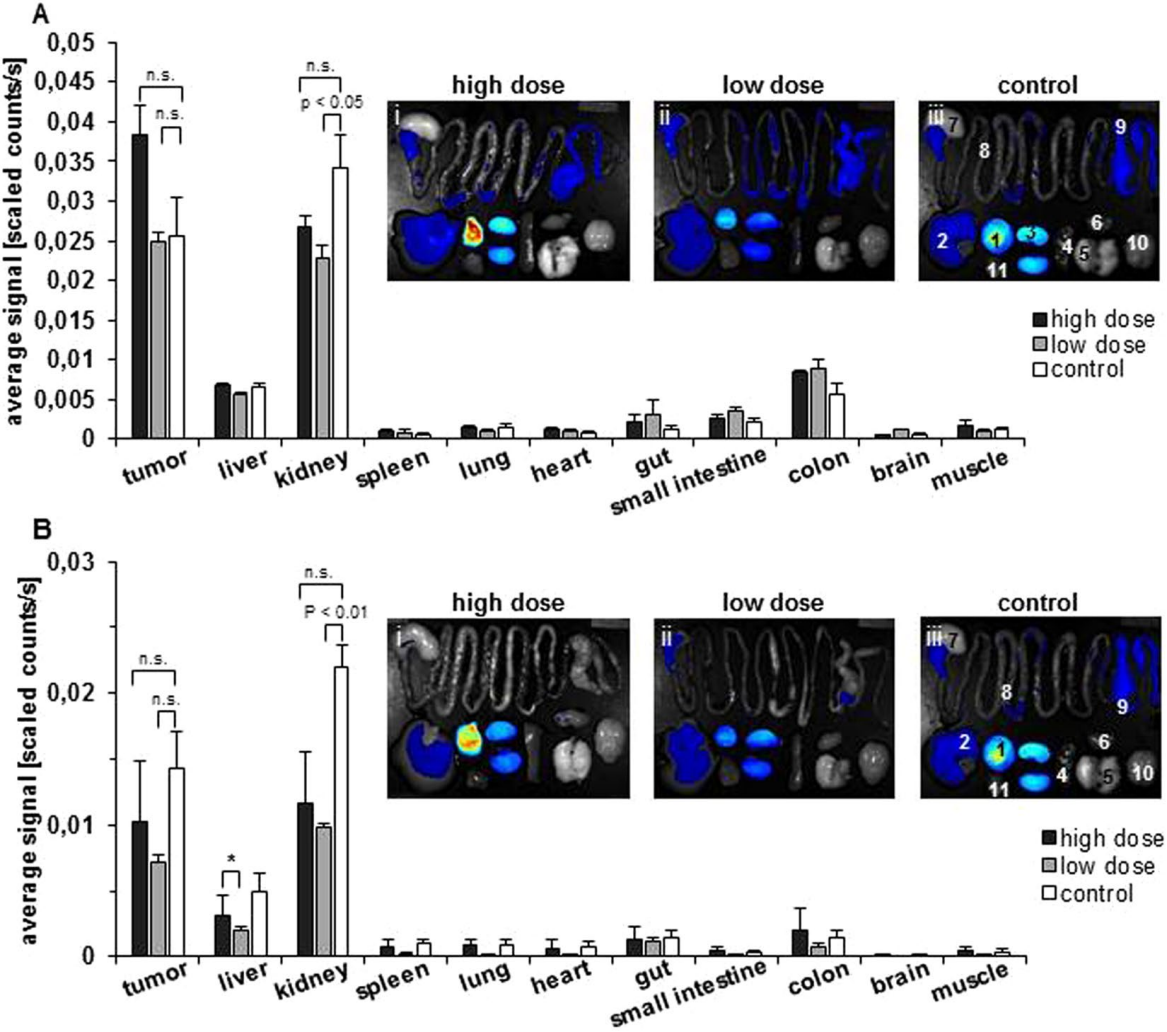
Biodistribution of the optical GRPR probe (BombesinRSense™ 680) and optical α_v_β_3_ integrin probe (IRDye^®^ 800CW RGD) at 8 days after hyperthermia treatment. (**A**) Semi-quantitative analysis of fluorescence intensities for GRPR in extracted mice organs after hyperthermia treatments. Macroscopic images with color-coded fluorescence intensities of the optical probe BombesinRSense™ 680 (2 nmol) from each treatment group. i: after high dose hyperthermia, ii: after low dose hyperthermia and iii: in untreated control. (**B**) Semi-quantitative analysis of fluorescence intensities for α_v_β_3_ integrin in extracted mice organs after hyperthermia treatments. Macroscopic images with color-coded fluorescence intensities of the optical probe IRDye^®^ 800CW RGD (1 nmol). i: after high dose hyperthermia, ii: after low dose hyperthermia and iii: in untreated control. Data represent the mean ± SEM of n = 5. (1 = tumor, 2 = liver, 3 = kidney, 4 = spleen, 5 = lung, 6 = heart, 7 = gut, 8 = small intestine, 9 = colon, 10 = brain, 11 = muscle), n.s.: not significant (p > 0.05).

*In vivo* experiments revealed a significant increase (p ≤ 0.05) of fluorescence intensities in tumors after administration either of a GRPR or an α_v_β_3_ integrin probe in tumors treated with high dose hyperthermia (46 °C, 8 days post treatment) compared to the non-treated control ones (Fig. 2). In contrast, in tumors treated with low dose hyperthermia these effects were less prominent. Untreated tumors showed no change of signal intensity for GRPR during the observation period. Representative color-coded images (Fig. 2C,D) illustrate the occurrence tumor fluorescence signals after hyperthermia treatments when optical probes targeting GRPR or α_v_β_3_ integrin were applied. Highest signal intensities for GRPR (Fig. 3) were mostly found at the tumor periphery 8 days post treatment.

### Biodistribution of GRPR and α_v_β_3_ integrin fluorescence probes in organs

The GRPR targeting probe accumulated mainly in the tumor and kidney (8 days post treatment). Interestingly, a distinct fluorescence was also seen in the kidney of untreated mice (Fig. 3A). In general, the probe fluorescence in the intestinal tract (gut, small intestine, and colon) and liver was not altered by hyperthermia treatment. No relevant fluorescence was seen in other organs (Fig. 3A). On the other hand, the α_v_β_3_ integrin targeting probe (IRDye^®^ 800CW RGD) accumulated mostly in the tumor and kidney (8 days either after hyperthermia, Fig. 3B). Interestingly, a distinct fluorescence intensity was also seen in the kidneys and the tumor of non-treated control animals.

### Hyperthermia affects tumor vascularization in a temperature dependent manner

**Figure 4:**
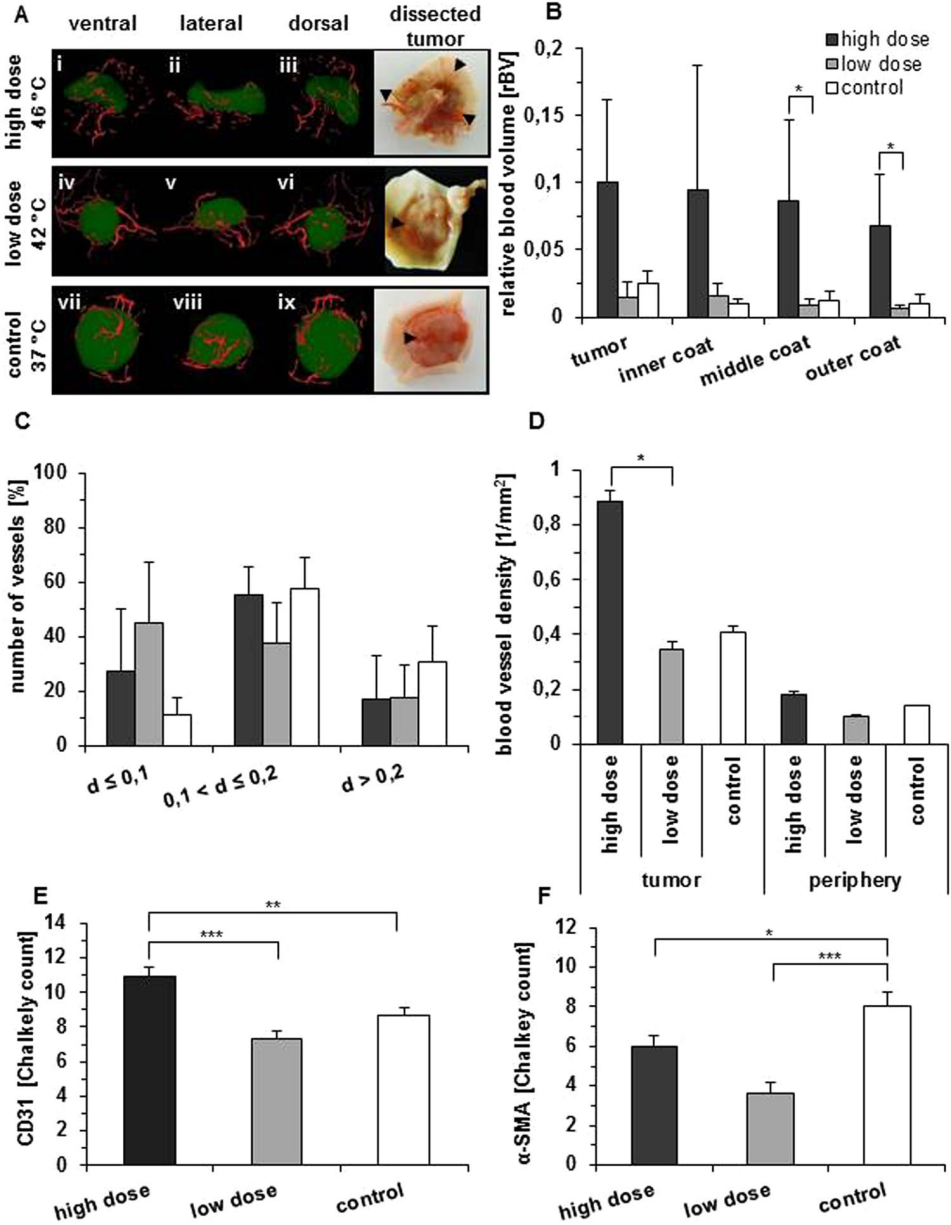
High dose hyperthermia influences the relative blood volume (rBV) and the relative blood vessel density (rBVD) within the tumor and its immediate environment. (**A**) Representative pictures of reconstructed µCT images. Red: vasculature, green: tumor tissue, arrowheads: vasculature. (**B**) Relative blood volume as estimated by the ratio of vascular volume of three standardized areas that surround the tumor and the tumor itself (for details see Methods). (**C**) Distribution of vessel diameters (d in mm) within the tumor. The number of vessels was related to the tumor volume. We defined three consecutive areas over the tumor surface, whereas each coat has a defined width of 0.592 mm (8 voxel). The total surface area which has been ﻿analyzed ﻿was of 1.776 mm (24 voxel) (see also Supplementary Fig. 5A and B). The vessel volumes of each area were calculated in proportion to the entire tumor volume. (**D**) Vessel density of the tumor and its periphery. Values were related to the cross sectional area of the tumor or periphery. The number of vessels in a defined field-of-view (FOV) was counted in randomly chosen digital slices of each tumor. The borders of the tumor and of the periphery were chosen to define the different FOV in each of the three planes (see Supplementary Fig. 5C). Therefore, each FOV was related to the respective tumor size. (**E** and **F**) Quantitative analysis of immunohistochemical staining of CD31 (**E**) or α-SMA (**F**) by Chalkley Count. Data are expressed as means ± SEM. Animals were treated either with low dose (42 °C for 1 h) or high dose hyperthermia (46 °C for 1 h). Controls were not subjected to any hyperthermia treatment. All data refer to 8 days post hyperthermia.

*Ex vivo* µCT imaging showed that the relative blood volume (rBV) in the tumor and its periphery, as an indicator for the vascularization grade, increased distinctly after high dose hyperthermia (8 days post hyperthermia, Fig. 4A,B). Additionally, both treated hyperthermia groups (high and low dose) showed a tendency for a distance-specific decrease of the rBV value from the tumor surface to its periphery. This pattern was not detectable in the control group. The increase of the rBV after high dose hyperthermia was accompanied by a change in the number of vessels and the distribution of their vessel diameter (Fig. 4C). In particular, the number of vessels with diameters larger than d > 0.2 mm decreased distinctly in the hyperthermia treated groups compared to the control ones. Furthermore, vessels with a small diameter increased. Especially low dose hyperthermia treated tumors exhibited mainly small vessels. High dose hyperthermia showed the highest vessel density in comparison to untreated controls (Fig. 4D). The histological semi-quantitative analysis revealed a significant (p ≤ 0.05) increase of tumor vascularization after high dose hyperthermia (Fig. 4E).Interestingly, the expression of smooth muscle actin (α-SMA), as indicator for the presence of mature endothelial cells, decreased after (high and low dose) hyperthermia (Fig. 4F).

### Immunohistochemistry corroborated the *in vivo* findings after hyperthermia treatment

**Figure 5:**
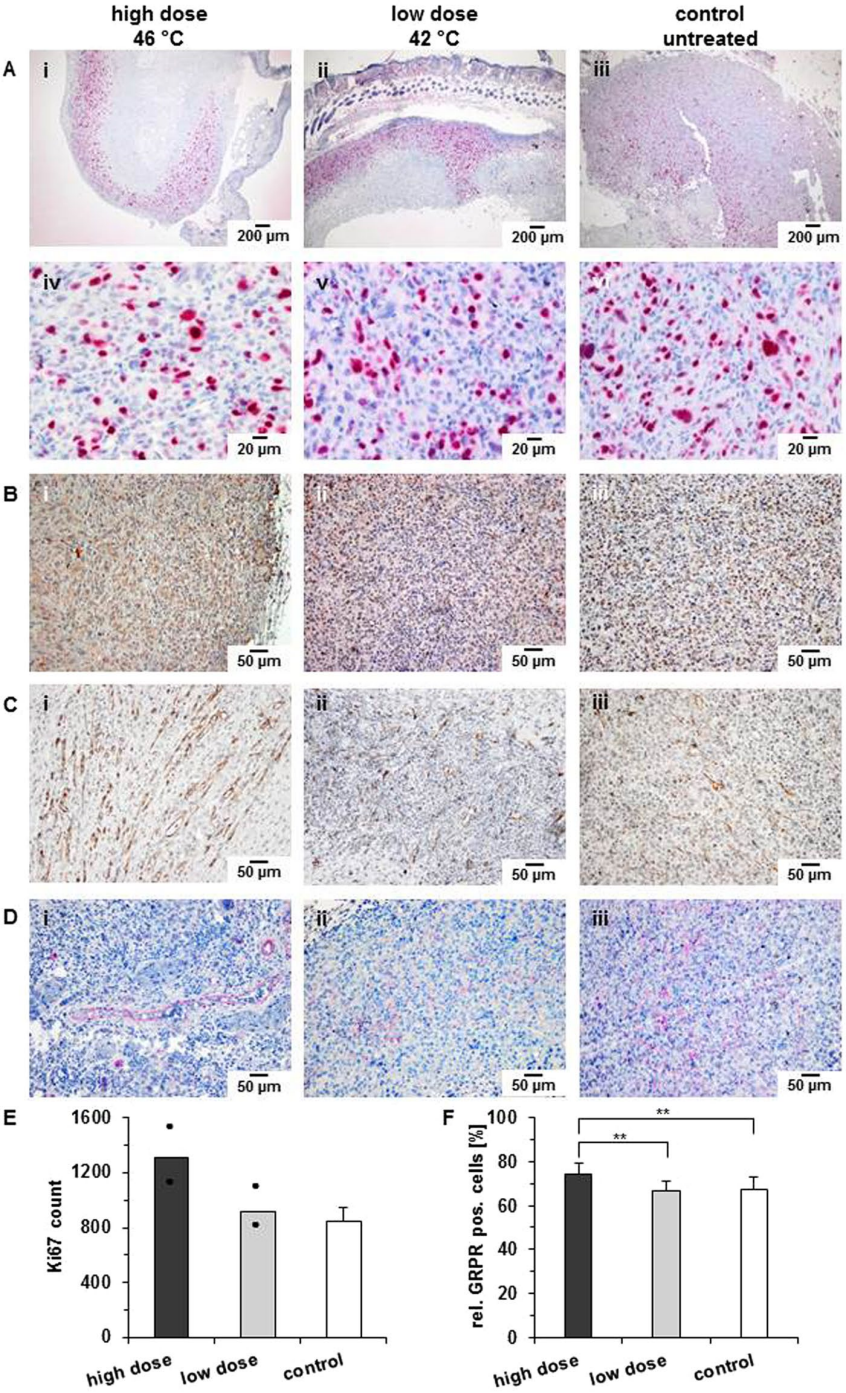
Hyperthermia impacts proliferation, GRPR-expression of tumor cells as well as the features of associated vessels. (**A**) Representative images of immunohistochemical staining of Ki67. Used magnification: 40 fold (i–iii) and 400 fold (iv–vi). (**B**) Representative images of immunohistochemical staining of GRPR. Used magnification: 200 fold (**C**) Representative images of immunohistochemical staining of CD31, magnification: 200 fold. (**D**) Representative images of immunohistochemical staining of α-SMA, used magnification: 200 fold. (**E**) Quantitative analysis of immunohistochemical staining of proliferation marker Ki67. (**F**) Analysis of immunohistochemical staining of GRPR by as proportion in relation to control (**p ≤ 0.01). Animals were treated either with low dose hyperthermia (42 °C for 1 h) or high dose hyperthermia (46 °C for 1 h). Controls were not subjected to any hyperthermia treatment. All data refer to 8 days post hyperthermia. For control of antibody reactivity see Supplementary Figure 6.

Low dose and high dose hyperthermia induced an intense and homogenous expression of Ki67 in the vital areas at the tumor margin (Fig. 5A). The amount of Ki67 positive cells was much higher in treated than in untreated tumors, and highest after high dose hyperthermia (Fig. 5A and E). The cell nuclei appeared to be enlarged after treatment. Untreated tumors showed a more heterogeneous Ki67expression pattern, since tumor areas of high expression alternated with those showing lower ones, and stained cells were less close to each other. In agreement with our *in vivo* findings, tumor cells treated with high dose hyperthermia revealed a significant increase of GRPR expression (Fig. 5B,F) and no alteration after low dose hyperthermia compared to controls. Additionally, staining of CD31 revealed vessel structures with a wider lumen in certain areas in tumors treated with high dose hyperthermia (Fig. 5C, i) compared to those treated with low dose hyperthermia or the non-treated controls. After high dose hyperthermia, α-SMA staining was diminished with exception of few areas with large blood vessels (Fig. 5D, i). α-SMA was highest in tumors of the control group (Fig. 4F) but evenly distributed (Fig. 5D, iii).

### Hyperthermia affected tumor volume in a temperature dependent manner

**Figure 6:**
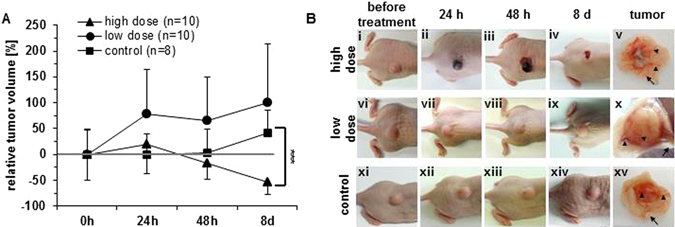
Low dose hyperthermia (42 °C) has no distinct impact on tumor volumes, whereas high dose hyperthermia (46 °C) induces tumor regression. (**A**) Tumor volumes after hyperthermia were normalized to the initial value prior to treatments. Data represented as mean ± SD (n = 8 or 10 mice for each condition). (**B**) Light images of tumor-bearing mice. The last panel on the right side represents dissected tumors with high degree of vascularization upon application of high dose hyperthermia (arrow-heads = tumor vasculature, arrows = skin). Controls were not subjected to any hyperthermia treatment.

After high dose hyperthermia tumors showed decreased volumes in relation to the initial value with ongoing time post therapy (Fig. 6A,B i–iv). The tumor volume of the high dose hyperthermia group was distinctly different from the non-treated control (p ≤ 0.001); this was not the case for the low dose hyperthermia group at least at 8 days post therapy (no significant differences with respect to controls, Fig. 6A). Interestingly tumors treated with low dosed hyperthermia showed an accelerated growth in comparison to the initially measured value (Fig. 6A). Non-treated controls revealed a comparably lower increase of the tumor diameter. After treatment, hemorrhages in the tumor area were macroscopically visible (Fig. 6B). Tumors showed an almost complete remission when treated with high dose hyperthermia (8 days post therapy, Fig. 6B). After dissection of treated tumors, irregularly shaped tumor tissue was visible; it was surrounded by a prominent vessel rim feeding the tumor. In comparison, the vasculature of non-treated tumors was macroscopically less prominent (Fig. 6B).

## Discussion

In this study we were able to show that (1) high dose hyperthermia (46 °C, 1 h) is capable of inducing both apoptosis and the expression of GRPR and α_v_β_3_ integrin in cells of the tumor region, (2) high dose hyperthermia *in vivo* reduces tumor volumes and concomitantly increases GRPR, α_v_β_3_ integrin, CD31, and Ki67 in these tumors, (3) high dose hyperthermia also changes the morphology of tumor vasculature by increasing the relative blood volume (rBV), the small-diameter vessel density, and by decreasing the expression of α-SMA, an indicator of the endothelial cell maturity in those tumors, (4) low dose hyperthermia (42 °C, 1 h) induces only moderate effects (i.e. on cellular apoptosis, GRPR, α_v_β_3_ integrin, CD31, and Ki67, α-SMA expression, rBV, small vessel density).

Interestingly, GRPR expression in isolated tumor cells was particularly prominent in those cell populations which were exposed to comparatively high temperature doses. The effect was accompanied by enhanced caspase-3 activation (cleaved caspase-3) which is a marker of irreversible cell damage and also for apoptosis^20^. This means that a distinct heat stress has been induced particularly with the high temperature dose protocol (46 °C, 1 h).

By applying a heating stimulus, more or less viable residual cells were still able to actively induce protein expression. The increased presence of GRPR after hyperthermia treatment was also observed in the *in vivo* situation in mice. Fluorescence signals of the GRPR were visible in tumors particularly at 8 days post treatment. These cells were able to express the proliferation marker Ki67, as immunohistochemistry analysis of tissues with high fluorescence revealed. Therefore, we assume that cells which managed to survive a strong heating stimulus are able to induce protein expression as response to thermal stress (see below).

The increase of GRPR was accompanied by an increase of α_v_β_3_ integrin expression in tumors, particularly after high dose hyperthermia. Therefore, these molecular structures might well be metabolically linked between each other as response to a heating stress. Namely, the impairment of the tumor vascularity due to hyperthermia leads to hypoxia, as a result of impaired nutrient supply. Hypoxia triggers HIF-1^21, 22^, which in turn promotes the expression of VEGF-1^23, 24^ as stress response reaction. VEGF was recognized to induce the expression and activation of EGFR^25, 26^ and GRPR^26^. Additionally, the activation of one of these receptors might affect the activation of other ones^27–30^. Such mechanisms could be one reason for the enhanced proliferation (as seen by the expression of Ki67 in tumor tissue samples) and increased presence of GRPR in residual tumor tissue after hyperthermia.

High dose hyperthermia seems to change the morphology of tumor vasculature as revealed by a high relative blood vessel volume and an increased blood vessel density compared to non-treated tumors. These observations are, again, signs for the induction of pro-angiogenic processes, as a result of the release of pro-angiogenic factors from residual tumor cells^31^ after the hyperthermic stress (see above). Our observations evidence that blood vessels are newly formed in treated tumors (increased presence of small vessels and reduced expression of α-SMA, a feature of mature blood vessels in the tumor region).

Irrespective of the encountered morphological features of tumor vascularity and protein expression, a reduction of tumor volumes was found after high dose hyperthermia (at least at 8 days post treatment). We associate such effects with loss of cells due to apoptosis and shrinkage of cells undergoing autophagy^32^. Autophagy, a process of cellular consumption, is thought to represent an energy conservation effort of cells which managed to escape from cell death. We postulate that heating can efficiently kill tumor cells but it might also leave some residual cells undergoing autophagy. They represent “dormant” cells in the tumor capable of recovering when the growth conditions have been restored^31^. The potential of recovery is underlined by the presence of protein expression (GRPR) and changed vascular morphology discussed above, together with the presence of cells expressing the proliferation marker Ki67. Such a response pattern is the manifestation of hormesis^33, 34^. As a result of hormesis, spreading of tumor cells with ongoing time is favored, particularly if one does not undertake any additional therapeutic treatments. Namely, it is known that an increased vascular density^35, 36^, hypoxia^37^ and a low expression of α-SMA^38, 39^ is beneficial for tumor cells to regrowth and metastasize. The same applies for the increased blood volume in tumors in conjunction with mitogenic and morphogenic effects of GRPR^40–42^, and finally the potential of GRPR to co-activate EGFR^27–30^ and VEGF^29^.

The fact that the effects on GRPR and α_v_β_3_ integrin expression in tumors were observed at 8 h post hyperthermia is related to the time the dormant tumor cells (in autophagy) need to trigger the pathophysiological pathways for metabolic restoration in the sense of “hormesis”. In particular, dormant cells “recognizing” hypoxic conditions have to induce the expression of relevant molecules such as VEGF-1, EGFR, GRPR, etc. (see above). This is a time-consuming process, which implicates the expression of new mRNA and protein molecules.

Interestingly, GRPR, α_v_β_3_ integrin, CD31, Ki67, and α-SMA expression were less prominent in the low dose hyperthermia group. Apparently, the applied heating stress was too low to induce remarkable heating responses. The fact that the tumor volume suddenly increased after 24 h after treatment might be ascribed to the induction of edema^43^ as a result of the permeabilization of the tumor vasculature without any morphological impairment. All these effects might represent a “mild” response to hyperthermia, by which less (or no) cells undergo autophagy and hormesis.

All in all, we attribute the enhanced expression of GRPR and α_v_β_3_ integrin to hormesis. Accordingly, we suggest the following strategy for hyperthermic treatments of tumors: a) particularly when using high dose hyperthermia, physicians should imperatively consider repeated and complementary therapy sessions together with an appropriate image-based therapeutic surveillance of recovery signs in short times intervals after therapy, b) low dose hyperthermia is attractive because it does not trigger “hormesis” as much as high dose hyperthermia. Hereto, repeated “mild” therapy sessions can stress cells for additional therapeutic modalities (chemotherapy, radiation) and should be applied together with a close-meshed image-based therapeutic monitoring. It might lead to smooth tumor eradications. In this context, changes of GRPR and α_v_β_3_ integrin expression could be used to identify a potential reorganization of tumor regrowth.

Interestingly, the hyperthermic treatment of tumors reduced the GRPR and α_v_β_3_ integrin optical probe accumulation in the kidneys. We postulate that the release of cell debris from the tumor to the blood as a result of the hyperthermic tumor treatment might lead to a systemic release of cytokines (e.g. IL-1, TNF-α, IL-6). It is widely known that cytokines reduce glomerular filtration rate but they can also be beneficial in ameliorating the host immune response against cancer cells, which has repeatedly been claimed in the literature^44^. Another, quite simple, explanation is clearance of probe metabolites, an issue which might be further clarified in future investigations.

The increased expression of GRPR and αVβ3 integrin might be beneficial for certain combination therapies targeting these proteins additionally to hyperthermia. For example, a GRPR inhibitor might have an effect on tumor cell proliferation, since GRPR stimulates tumor growth^45^. Its inhibition might also increase the effect of hyperthermia in analogy as it has already been with chemotherapy^46^. Furthermore, α_v_β_3_ integrin is known to play an important role in adhesion of newly formed endothelial cells in the tumor extracellular matrix. Its inhibition might reduce migration of endothelial cells^47^, and prevent proliferative kinase cross talks (e.g. with the extracellular regulated kinase (ERK1/2)^48^.

In this study we gave first evidence that GRPR and α_V_β_3_ integrin expression might be used to assess reorganization of tumors as consequence of an adaptive response (hormesis) of dormant (authopagic) cells to the heat stress stimulus. Such effects have to be considered more in detail in future. In this sense, we shed more light into the question why tumors treated at temperatures above the widely recognized thresholds previously established from *in vitro* experiments^49, 50^ are still able to proliferate. In the *in vivo* situation, the response to a heating stimulus has a higher degree of metabolic and physiological complexity, as discussed above. In this context, hyperthermia is a subject of molecular and personalized medicine, as it has been claimed for other oncological modalities as well.

## Conclusion

We have shown that high dose hyperthermia induces a strong response of the tumor tissue in terms of an overexpression of GRPR and changed vascular morphology; it is a process, which we identified by the presence of α_v_β_3_ integrin, the relative blood volume (rBV), the small-diameter vessel density, and decreased expression of α-SMA. The strong response was suggested to be associated with “dormant” residual cells in the tumor region capable of organizing recovery. By utilization of lower treatment temperatures, these effects were less prominent. This distinct response pattern might well be an explanation why the therapeutic outcome of hyperthermia in the clinic has been so heterogeneous until now. Consequently, we propose to consider: (a) repeated high dose hyperthermia together with complementary therapy sessions or (b) to use repeated “mild” hyperthermia sessions in order to stress tumor cells for additional therapeutic modalities (chemotherapy, radiation) and c) to also reconsider the presently established thresholds for temperature dosages and therapeutic strategies when treating tumors via hyperthermia as an adjunct to current oncologic modalities. This means that the parameters GRPR, α_v_β_3_ integrin expression, and changed vascular morphology do reflect potential regrowth features of tumors after hyperthermia treatment. They might be exploited for an image-based therapeutic monitoring of hyperthermia in the future.

## Materials and Methods

### Cell-line and culture conditions

Breast adenocarcinoma cell line MDA-MB-231 (CLS Cell Lines Service) was cultured in DMEM Ham’s F12 (1:1) (Gibco) containing 5% FCS (Gibco) at 37 °C in a humidified 5% CO_2_ atmosphere. In a previous experiment, the cells were shown to be able to express GRPR (Suppl Fig. 1).

### Hyperthermia treatment of cells

To differentiate temperatures having only transient or lethal cytotoxic effects, cells were seeded 24 h prior to treatment in a culture flask. To exactly measure the temperature during hyperthermia (hot air, incubator), a fiber-optic thermocouple (OPTOCON AG) was used. The duration of treatment was 1 h. Immediately after hyperthermia, cells were incubated at standard conditions. An untreated control maintained at standard culture conditions (37 °C, 5% CO_2_, humidified air).

### Flow cytometry to assess presence of GRPR and activation of caspase-3

After external (hot air) hyperthermia, cells were harvested and washed in ice-cold phosphate buffered saline (PBS). The cells were fixed in 2% formaldehyde and permeabilized with 0.1% Triton X-100 (Sigma-Aldrich). The GRPR and cleaved caspase-3 were stained indirectly using a FITC-conjugated anti-rabbit IgG secondary antibody (abcam). The following primary antibodies were used: The rabbit-anti-GRPR antibody (antibodies-online Inc.) and the monoclonal rabbit-anti-cleaved caspase-3 antibody (Cell Signaling Technology). The measured values were normalized to untreated control cells, seeded, harvested, and stained at the same point of time. Isotype, negative, and positive controls were used (see Supplementary Figure 2 for more information).

### Western Blot to assess GRPR expression

After hyperthermia treatment, cell lysates were washed in ice cold PBS and prepared by homogenization in RIPA buffer [50 mM TRIS-HCl pH 8.0, 150 mM NaCl, 1% NP-40, 0.5% Na-desoxycholate, 0.1% SDS, 1 mM NaF, 1 mM DTT, 0.4 mM PMSF, 0.1 mM Na_3_VO_4_] supplemented with protease (Complete^TM^) and phosphatase inhibitor cocktail (Roche Diagnostics). Protein concentration was determined using the Bradford assay. Protein loads of 5 µg were resolved by SDS-PAGE and analyzed by immunoblotting. The following primary antibodies were used: anti-GRPR antibody (antibodies-online) and anti-α-tubulin antibody (Boehring). The second antibody was a polyclonal HRP labelled goat-anti-rabbit IgG (Santa Cruz Biotechnology). Isotype, negative, and positive controls were used (see Supplementary Figures 3 and 4 for more information).

### Animals and tumor model

All experiments were in accordance with international guidelines on the ethical use of animals and were approved by the regional animal care committee (No. 02-068/11). During experimentation, the animals were anesthetized with 2.0–2.5% Isoflurane (Actavis). For tumor implantation, 2 × 10^6^ cells were suspended in 100 µl Matrigel™ (BD Biosciences) and implanted subcutaneously in the middle of the back of the animals.

### Hyperthermia of tumors in mice

*In vivo* external hyperthermia treatments were performed via exposure of tumors to hot water (n = 8). During low dose hyperthermia, the tumor surface exhibited a temperature of 42 °C, and a minimum of 40 °C in the inner tumor tissue; during high dose hyperthermia, temperatures of 46 °C were reached at the tumor surface and at least 44 °C in the inner tumor area. The hyperthermia treatment was conducted for 1 h. The tumor and body temperatures were monitored using fiber-optic thermocouples (OPTOCON AG). These preconditions were determined in a prototype experiment. Tumor size was measured 24 h, 48 h, and 8 days after hot water hyperthermia. The tumor volume was calculated by the formula V = π/6 × length × width × height of the tumor^51^.

### *In vivo* imaging of GRPR and tumor vascularization after hyperthermia

The intravenous injection of 1 nmol IRDye^®^ 800CW RGD and 2 nmol BombesinRSense™ 680 per mouse was done simultaneously at 24 h and 7 days after treatment in order to image change of tumor vascularization, probe accessibility after treatment and GRPR expression. 24 h after administration of the optical probes, the fluorescence intensities were monitored by whole body near infrared fluorescence (NIRF) imaging system (Cri Inc.) using the filter “Deep Red” (excitation filter: 670-710 nm, emission filter: 750 nm) for IRDye^®^ 800CW RGD, and the filter “Yellow” (excitation filter: 570–610 nm, emission filter: 645 nm) for BombesinRSense™ 680. Comparatibility of fluorescence intensity of each image was attained by normalization to scaled counts per second. Each contrast agent has been analyzed separately for its fluorescence spectrum after subtracting the spectrum of autofluorescence. The contrast of each contrast agent was determined through the tumor-to-muscle-ratio (TMR), i.e. by dividing the average fluorescence signal of a defined region of interest (ROI) in the tumor area and the fluorescence signal of normal tissue (ROI,﻿ muscle of the hint legs). The TMR for each optical probe and the point of time after hyperthermia were related to the native images (without contrast agent) in order to exclude natural intrinsic contrast at the wavelengths of interest. Contrast agent accumulation was compared to the non-treated control group (n = 10).

### Biodistribution of optical probes/*Ex vivo* optical imaging

At 8 days after treatment, the fluorescence intensity of dissected organs was measured under the same instrument settings as described for *in vivo* optical imaging.

### Analysis of tumor vascularization via µCT

Immediately after killing, the mice were prepared for vessel perfusion according to the surgery guidelines of Gage and Kipke *et al*.^52^. In this context, the mice were perfused with 10 ml of the lead-based silicon rubber casting resin Microfil^®^ MV-130 and MV-diluent, prepared according to the manufactor’s guidelines (Flow Tech). All scans were performed using a dual source µCT cone-beam scanner TomoScope^®^ Synergy (CT Imaging). Scans were visualized and analyzed using Imalytics^®^ preclinical software (Philips Research) after three-dimensional volume rendering of reconstructed high-resolution µCT data sets. In order to analyze the tumor-associated vessels located in the immediate periphery of the tumor, we defined three consecutive areas over the tumor surface, whereas each coat has a defined width of 0.592 mm (8 voxel). The total surface area of analysis was of 1.776 mm (24 voxel) (Supplementary Fig. 5A,B). The volumes of the respective vessels of each area were calculated in proportion to the entire tumor volume. To calculate the relative blood vessel density (rBVD), the number of vessels in a defined field-of-view (FOV) was determined. Therefore, three randomly chosen digital slices in each tumor were evaluated. The borders of the tumor and of the periphery were chosen to define the different FOV in each of the three planes (Supplementary Fig. S5C). Therefore, each FOV was related to the respective tumor size.

### Immunohistochemistry

Tissue sections were either blocked with avidin/biotin or with Peroxidase Block (Dako). The following antibodies were used: For detection of Ki67 primary antibody was a monoclonal anti-rabbit-Ki67 antibody (abcam) and isotype control a monoclonal rabbit IgG (abcam) (primary antibodies). For detection of GRPR: monoclonal rabbit-anti-human-GRPR-antibody (antibodies-online Inc.), a polyclonal rabbit IgG (h & l) antibody (Thermo Scientific) (isotype control). CD31: polyclonal rabbit anti-CD31 antibody (abcam), rabbit polyclonal IgG (abcam) as isotype control. α-SMA: monoclonal mouse anti α-SMA (abcam) as primary antibody and Mouse IgG2a (BIOZOL Diagnostica) as isotype control. The detection of the markers was performed either with addition of streptavidin-alkaline phosphatase or peroxidase labelled system (both from Dako). For tissue counterstaining, nuclear structures were stained with Mayer’s Hematoxylin (Fluka/Sigma-Aldrich). Quantification was performed using the software cellSens (Olympus). Positive, isotype, and negative controls of immunostaining were used (Supplementary Figure 6 for more information).

### Statistical analyses

Data are expressed as means ± SEM or standard deviation respectively. Statistical significance was analyzed by Mann-Whitney. A p-value of 0.05 or less was considered statistically significant. The number of parallels or animals in the experiments is given in the figure legends.

## Electronic supplementary material

Supplementary Information
