# Supplementary material for: How gastrin-releasing peptide receptor (GRPR) and αvβ3 integrin expression reflect reorganization features of tumors after hyperthermia treatments
Source: Sci Rep. 2017 Jul 31;7:6916. doi: 10.1038/s41598-017-06100-7 (PMC5537297; doi:10.1038/s41598-017-06100-7)
Supplement: Supplementary file 1 — Supplementary Information [file 41598_2017_6100_MOESM1_ESM.doc]

**Supplementary Figures**

**How gastrin-releasing peptide receptor (GRPR) and αvβ3 integrin expression reflect regrow features of tumors after hyperthermia treatments**

Sandra Hallasch, cand.med.^1^; Sindy Frick, Ph.D^1^, Maximilian Jung, BS^1^, ^2^, Ingrid Hilger Ph.D^1^

^1^ Institute of Diagnostic and Interventional Radiology, Department of Experimental Radiology, Jena University Hospital – Friedrich Schiller University Jena, Am Klinikum 1, 07747 Jena, Germany

^2^ Department of Medical Engineering and Biotechnology, University of Applied Science Jena, Carl-Zeiss Promenade 2, 07745 Jena, Germany

Corresponding author: Prof. Dr. Ingrid Hilger, Institute for Diagnostic and Interventional Radiology, Jena University Hospital – Friedrich Schiller University Jena, Forschungszentrum Lobeda, Am Klinikum 1, D-07747 Jena, Germany. Phone: 0049-(0)3641-9325921, Fax: 0049-(0)3641-9325922, e-mail: ingrid.hilger@med.uni-jena.de


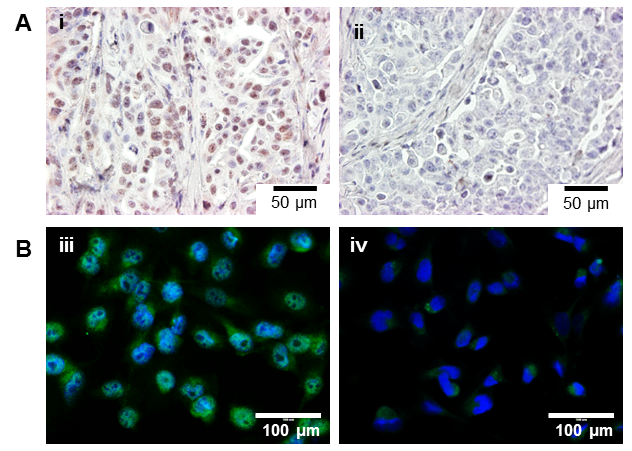


***Supplementary Fig.1. The breast cancer cell line MDA‑MB‑231 is able to express GRPR.*** A) Immuohistchemistry of paraffin embedded MDA‑MB‑231 non‑treated tumors show expression of GRPR. (i: Brown stained areas indicate expression of GRPR, ii: The conducted no‑primary control showed no staining for GRPR.) B) Immunofluorescence detection of GRPR receptor in untreated MDA‑MB‑231 cells (i: GRPR antibody, ii: isotype control). From the methodological point of view: Cells were fixed with 2 % formaldehyde (Carl Roth) and permeabilized with 0.1 % Tween (Sigma-Aldrich). The anti-GRPR-antibody (antibodies-online) was added and incubated overnight at +4 °C. The secondary antibody was a FITC‑labelled IgG (H+L) (Abcam) antibody. As control for specificity of the primary antibody a rabbit IgG isotype control (Thermo Scientific) was conducted in each experiment. The counterstaining was performed with Hoechst 33258 bisbenzimid (Applichem).


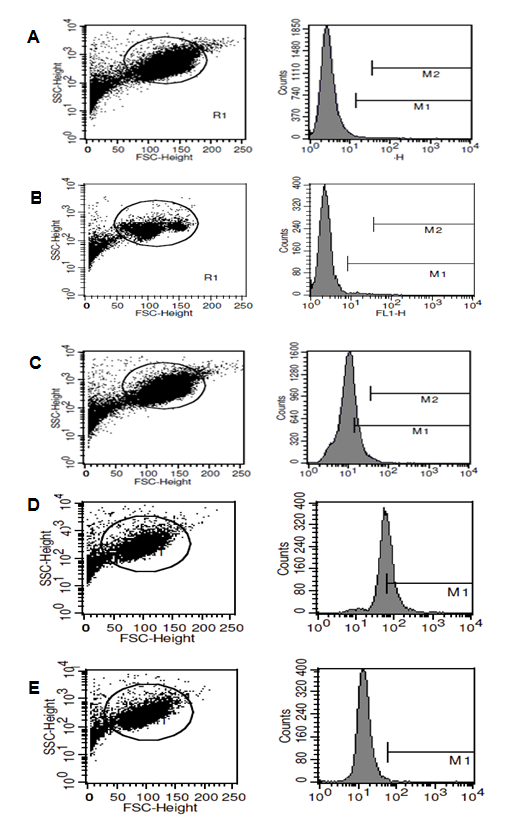


***Supplementary Fig. 2. Detection of GRPR and Caspase via FACS on MDA-MB-231 cells as shown in Figure 1A is specific***. A) Negative control of MDA-MB-231. B) No primary control, C) GRPR and caspase-3 isotype control (both rabbit anti-human), D) GRPR positive control on HT-29 cells and E) corresponding no primary control on HT-29 cells.


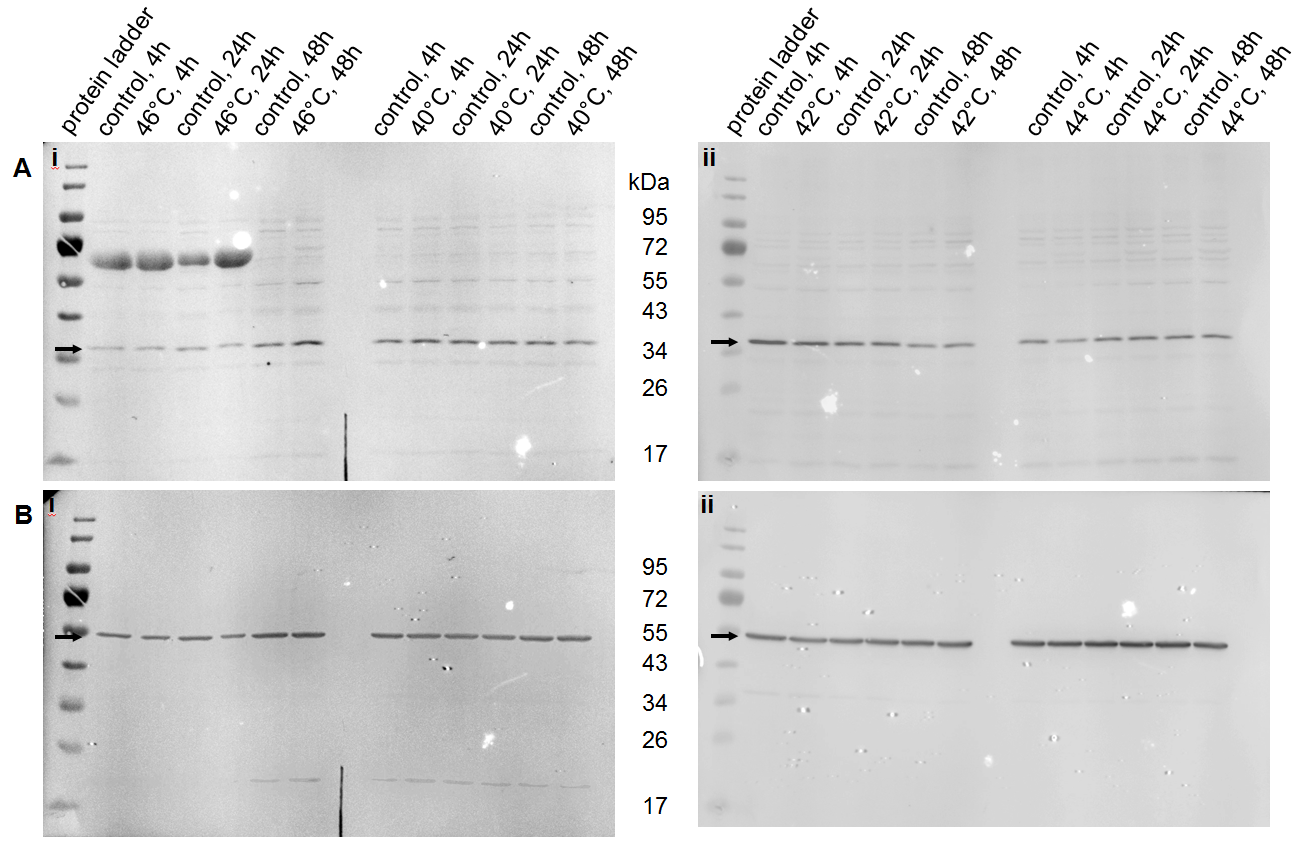


***Supplementary Fig. 3.*** ***Full length Western blots depicted in Figure 1B.*** Representative Western blots for GRPR expression (37 kDa, A) and α-Tubulin (55 kDa, B) after 1 h of hyperthermia treatments at temperatures ranging from 40 °C to 46 °C. B). i) Temperature treatments at 46 °C and 40 °C with respective controls on the same blot, ii) temperature treatments at 42 °C and 44 °C with respective controls on the same blot. Chemiluminescence of protein bands of all blots was acquired using the same settings. Arrows mark the estimated molecular weight of the protein band of interest.


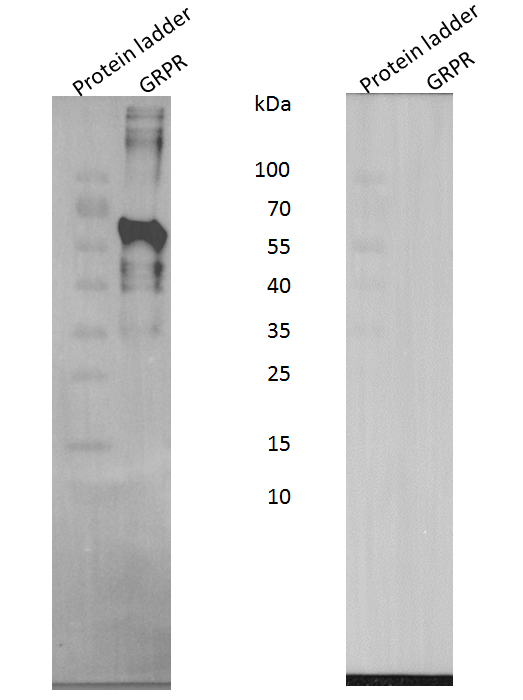


***Fig. S4. Full length control Western blots depicted in Figure 1.*** Left: positive control using GRPR ( protein nennen). Right: no primary control. The difference of detected molecular weights between the positive control (synthetic protein) and the lysates (isolated protein in Figure 1) are attributed to the isolation procedure of the membrane protein GRPR, by which parts of the protein might be gotten lost due to its amphipilic nature of the different domains (extracellular, transmembrane and intracellular).


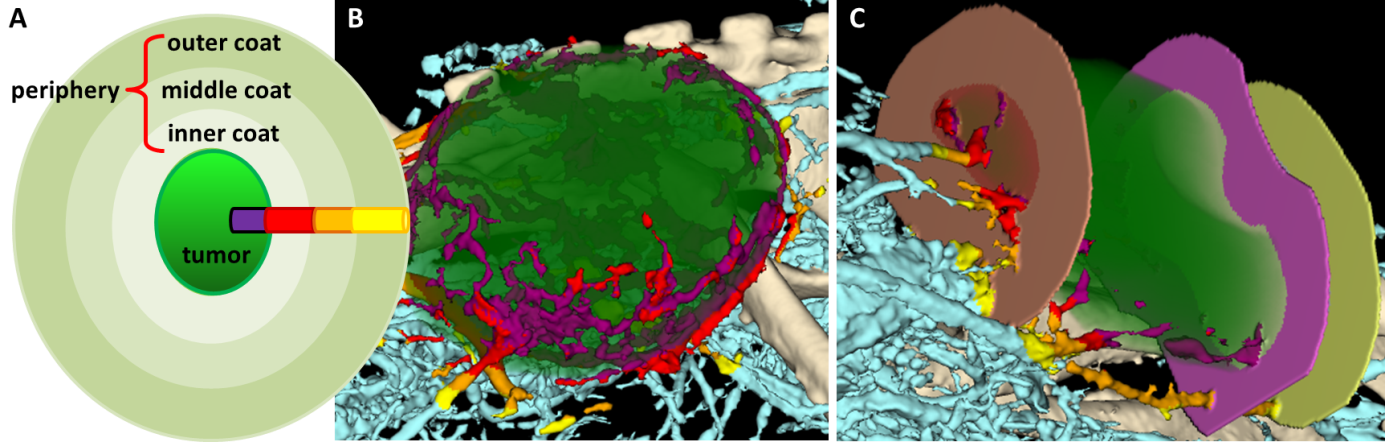


**B**

**C**

**outer coat**

**middle coat**

**inner coat**

**tumor**

**periphery**

**A**

***Supplementary Fig. 5. Reconstruction of the tumor and its surrounding tissue with the software “Imalytics”.*** A) Schematic representation of the tumor and the consecutive layers. The vessels lying inside the coats were defined as periphery vessels. B) The actual depiction of the tumor and its vascular system with the software “Imalytics”. Vessels which were not part of the analysis are displayed in light blue. C) Tumor with three added planes, each consisting out of two FOVs, one inside the tumor, one in its periphery. The vessel density was calculated with the number of vessels crossing the FOVs and the respective surface of the latter.


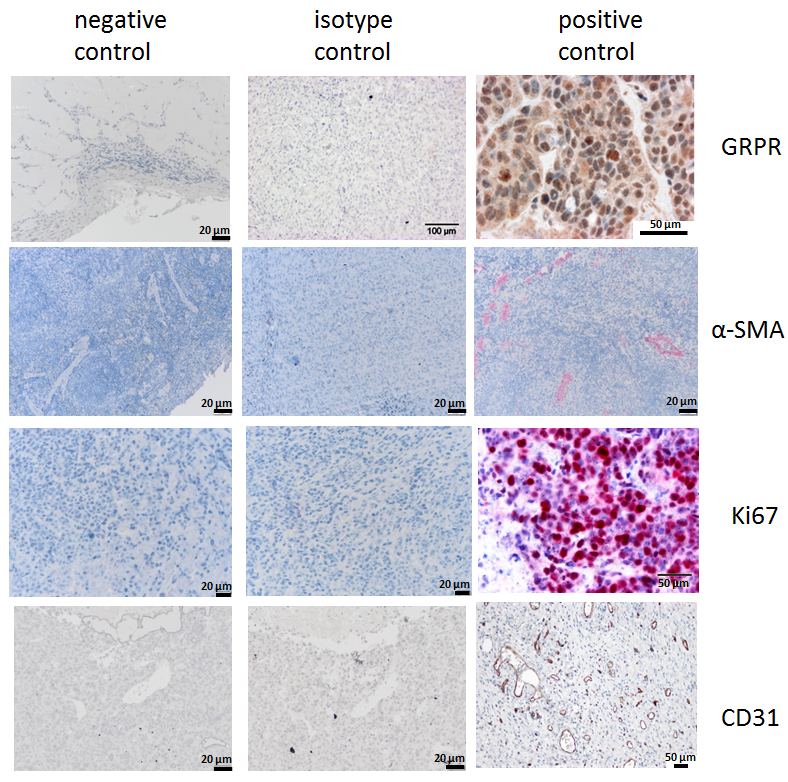


***Supplementary Fig. 6. Immunohistochemistry staining in Figure 5 is specific***. GRPR: positive control is HT29 tumor tissue according to Rick and Buchholz^1^; α-SMA: positive control is spleen tissue, Ki67: positive control is A431 tumor according to Brunetto de Farias et al.^2^; CD 31: the positive control is represented by large blood vessels in MDA-MB-231 tumors with a clear lumen.

**References**

1 Rick, F. G. *et al.* Combination of gastrin-releasing peptide antagonist with cytotoxic agents produces synergistic inhibition of growth of human experimental colon cancers. *Cell Cycle* **11**, 2518-2525, doi:10.4161/cc.20900 (2012).

2 Brunetto de Farias, C. *et al.* Reduced NGF Secretion by HT-29 Human Colon Cancer Cells Treated with a GRPR Antagonist. *Protein & Peptide Letters* **16**, 650-652, doi:10.2174/092986609788490177 (2009).
